# Discrepancies in Perceived Indulgent Parenting, Relationship Satisfaction, and Psychological Well-Being of Adolescents and Parents

**DOI:** 10.3390/children11040393

**Published:** 2024-03-26

**Authors:** Qinglan Feng, Ming Cui

**Affiliations:** Department of Human Development and Family Science, Florida State University, Tallahassee, FL 32306, USA; qfeng@fsu.edu

**Keywords:** adolescence, indulgent parenting, relationship satisfaction, psychological well-being, intraclass correlation (ICC)

## Abstract

Indulgent parenting has been associated with adolescents’ psychological well-being problems; however, prior research has primarily relied on adolescents’ report of such parenting behavior and its association with their own well-being, often overlooking parents’ perceptions of indulgence and their own well-being. In this study, we address this gap in the literature by examining the agreement and disagreement between parents’ and adolescents’ perceptions of indulgent parenting and the implications for the psychological well-being of both adolescents and their parents. Further, we explore the role of adolescent–parent relationship satisfaction as a potential factor affecting these associations. Our investigation was based on data from 128 parent–adolescent dyads. Utilizing structural equation modeling with double-entry intraclass correlations (ICC_DE), our analyses revealed several main findings: (1) adolescents perceived higher levels of indulgent parenting than their parents did; (2) disagreement in perceived indulgent parenting between parents and adolescents was linked to psychological well-being problems for both adolescents and their parents; and (3) disagreement in perceptions in indulgent parenting had a stronger association with adolescents’ well-being problems when adolescents reported greater relationship satisfaction with their parents. These findings provide insights into perceptions of indulgent parenting within parent–adolescent relationships and bring psychological implications for both adolescents and their parents.

## 1. Introduction

Adolescence is a critical developmental stage when many changes happen, which could be challenging for both adolescents and their parents. As children become adolescents, they face changes brought on by the process of puberty, increasing academic demands, and growing social pressure. Lerner and Steinberg [1] suggest that these challenges, though accompanied by opportunities, could lead to psychological well-being problems among adolescents. As a result, parenting and parent–adolescent relationships need to be renegotiated during adolescence. Given the developmental challenges faced by adolescents, indulgent parenting, a particular type of parenting behavior high in parental responsiveness but low in demandingness, could put adolescents at risk of well-being problems (e.g., [2,3]). Further, adolescents and their parents may have different perceptions regarding such indulgent parenting behavior; they may disagree with their perceptions, which could result in well-being problems among adolescents (e.g., [4]). In addition to adolescents’ well-being, the demands of parenting and related disagreements could also bring well-being problems among parents (e.g., [5]). Furthermore, parent–child relationship satisfaction may affect the relationship between perceptions of indulgence and the well-being of adolescents and their parents (e.g., [6]). To address these issues, the purposes of this study are to investigate (1) the potential discrepancies between adolescents and their parents in their perceptions of indulgent parenting, (2) the associations between these discrepancies in perceived indulgent parenting and well-being problems among adolescents and their parents, and (3) the role of parent–adolescent relationship satisfaction in these associations. 

### 1.1. Indulgent Parenting and Well-Being Problems of Adolescents and Parents

Parenting plays an essential role during adolescence because it provides adolescents with the necessary guidance, emotional support, and structure with clear boundaries to navigate challenges successfully. Psychosocial development theory [7] proposes that, during this stage, adolescents seek identity, develop the concept of self, and pursue independence from their parents. Among the many tasks during adolescence, Havighurst’s concept of developmental tasks also suggests adolescence to be a period to achieve emotional independence from their parents [8]. Self-determination theory [9] also addresses adolescents’ growing psychological needs for autonomy, competence, and relatedness, calling for the need for corresponding changes in parenting and parent–child relationships. As one of the four parenting styles proposed by Baumrind [10], indulgent parenting is characterized as a style in which parents are very responsive to their children’s wants and needs but tend to be less demanding in enforcing rules and setting limits. As a result, indulgent parenting during adolescence is often considered developmentally unsuitable because it could deprive adolescents of the opportunities to explore and assume responsibilities, ultimately leading to well-being problems [9]. 

Although there have been some inconsistent findings, previous studies have generally supported these theories and indicated that indulgent parenting has negative effects on adolescent development (e.g., [11,12,13]). Faced with the challenges of parenting adolescent children, parents of adolescents who practiced indulgent parenting have also been found to report high levels of well-being problems [4].

### 1.2. Disagreement in Indulgent Parenting and Adolescents’ Well-Being Problems

Disagreement in perceptions of indulgent parenting between adolescents and their parents could reflect different attitudes and beliefs between adolescents and their parents regarding indulgent parenting. From the perspective of adolescents, indulgent parenting may be viewed as more negative compared to their parent’s perspective, as such parenting may fail to fulfill adolescents’ evolving psychological needs for seeking independence and responsibility [14]. Adolescents, therefore, might interpret indulgent parenting as their parents’ underestimation of their capability to handle things independently and to take on responsibility, resulting in feelings of frustration, stress, and other adverse psychological outcomes, especially when their perceptions clash with those of their parents. On the other hand, parents, who may be unaware of their adolescent children’s changing psychological needs, may hold different beliefs about parenting compared to their adolescent children, which can lead to differing perceptions of indulgent parenting between parents and adolescents. The discrepancies in perceptions, rather than the absolute levels, of indulgent parenting, may cause problems in the well-being of adolescents. 

The concept of goodness of fit [15,16] provides support for the association between discrepancies in adolescents’ and parents’ perceptions and adolescents’ well-being problems. Adolescents’ demands change in parenting with their increasing needs for independence and autonomy. Developmentally appropriate parenting needs to be renegotiated between the adolescents and their parents to achieve the “goodness of fit”. The concept of goodness of fit supports that parent–adolescent dyads may have varying perceptions of indulgent parenting, and that a low fit between parenting and adolescent development could produce negative effects on adolescents’ development and adjustment [17]. During this developmental stage, adolescents often seek greater autonomy and independence from their parents. Parents, however, may not always recognize these evolving needs during the transition from childhood to adulthood and may continue to expect their adolescent children to rely on them. This discrepancy in expectations could result in ineffective parent–child interactions, impacting adolescents’ well-being. Additionally, the stage–environment fit theory [18] underscores the significance of alignment or “match” between adolescents and their parents in terms of their evolving roles and expectations during this critical phase of development. As a result, the mismatch or imbalance between the perceptions of indulgence practices by adolescents and their parents and the need for renegotiation between the developing adolescents and their parents, as reflected by discrepancies in perceptions of indulgent parenting, could bring negative consequences on the well-being of adolescents. 

Empirical evidence on parenting has documented systematic differences between parents’ and their children’s reports of parenting behaviors [19,20]. A meta-analysis of 85 studies investigating the congruence of parents’ and children’s perceptions of parenting found distinct patterns of disagreement [21]. For instance, children tended to report higher levels of psychological control than their parents did. Furthermore, studies have identified that discrepancies in parent–child reports of parenting, including parental warmth, hostility, and inconsistent discipline, were related to children internalizing problems, such as anxiety and depression [22]. These findings underscored the importance of considering the unique perspectives of both parents and adolescents in understanding the impact of parenting behaviors on the psychological well-being of adolescents.

Few studies have investigated discrepancies in perceptions of indulgent parenting in particular between parents and adolescents. The studies on how such discrepancies were related to adolescents’ well-being were even more limited. Further, the majority of studies examining the relationship between indulgent parenting and adolescent development have relied on the single-informant method, with only a few incorporating reports from both adolescents and their parents. Veldorale-Griffin et al. [4] conducted a study on indulgent parenting with reports from adolescents and their parents. Their findings revealed that adolescents perceived fewer rules compared to their parents’ perceptions. They also found that, for adolescents, parental material indulgence was linked to lower levels of life satisfaction, mediated through increased stress. However, they analyzed reports from parents and adolescents separately and did not explore the role of disagreement in indulgent parenting between adolescents and their parents and its potential impact on the well-being of adolescents. 

Feng and Cui [23] investigated indulgent parenting and its relationship with psychological well-being with parents’ and adolescents’ reports. They found that adolescents’ (but not their parents’) perceptions of parental indulgence were associated with their well-being problems. This study, though it used a dyadic approach, did not examine disagreement. Love et al. [24] conducted a study involving emerging adult children and their parents and reported that emerging adult children perceived higher levels of indulgent parenting than their parents did and that disagreement in these perceptions was associated with emerging adult children’s depressive symptoms. While this study examined disagreement in indulgent parenting, the sample consisted of emerging adult children. Nevertheless, these studies offered insights into the potential discordance between adolescents and their parents in their perceptions of indulgent parenting and its potential implications for the well-being of adolescents.

### 1.3. Considering Parents’ Well-Being Problems

Family systems theory [25,26] posits that a family unit constitutes an intricate social system in which members share deep emotional connections. Changes in one family member’s functioning can reverberate throughout the entire system. This theory highlights that the effects of parenting could extend beyond adolescent children to also affect the parents as well. Parenting stress theory [27] suggests that the daily challenges associated with parenting can act as stressors, potentially affecting parents’ well-being by inducing parenting-related stress. Indulgent parenting, characterized by high attentiveness coupled with tolerance for misbehavior, may contribute to increased parenting stress, subsequently impacting parental well-being [28]. Furthermore, in line with family systems theory, disagreement between adolescents and their parents may disrupt family organization and cohesion, thereby negatively affecting the functioning and well-being of each family member [29]. Very few studies have examined parents’ well-being related to indulgent parenting behaviors or perceptions. In Veldorale-Griffin et al.’s [4] study, they reported that behavioral indulgence was associated with lower life satisfaction among parents of adolescents through elevated stress levels. Feng and Cui [23] reported that parents’ perception of indulgent parenting was related to their own psychological problems.

### 1.4. The Role of Parent–Adolescent Relationship Satisfaction

Research has consistently shown a positive association between parent–adolescent relationship satisfaction and adolescents’ psychological well-being (e.g., [6,30]). A satisfying parent–child relationship could function as a protective factor, promoting a positive effect or buffering an adverse effect of parenting on adolescents’ psychological well-being. The role of relationship satisfaction in the relationship between discrepancies in parent–adolescent perceptions of indulgent parenting and the psychological well-being of adolescents and their parents, however, remains unexplored. From a family systems perspective, it is plausible that individuals who are satisfied with their relationship with another family member place more value on their communications and interactions; therefore, disagreement in their perceptions could carry more emotional weight on them and cause more stress in the context of their close relationship. Given the commonly found protective effects of a satisfying parent–child relationship in child development, the potential source of stress within a close relationship when facing disagreement underscores the need for empirical studies to delve deeper into these complex associations.

### 1.5. The Present Study

To address the gaps in the current literature and to advance the understanding of indulgent parenting and the psychological well-being of adolescents and their parents, we propose three objectives in this study. The first objective is to examine discrepancies between adolescents’ and their parents’ perceptions of indulgent parenting. The second objective is to assess the association between disagreement in their perceptions and the psychological well-being problems of both adolescents and their parents. The third objective is to investigate whether parent–adolescent relationship satisfaction affects these associations.

Building on theories and research, we proposed three corresponding hypotheses. First, adolescents and parents would perceive different levels of indulgent parenting, with adolescents reporting higher levels (H1). Second, discrepancies in perceptions of indulgent parenting would be positively associated with psychological well-being problems for both adolescents and their parents (H2). Finally, parent–adolescent relationship satisfaction would moderate the relationship between parent–adolescent disagreement in indulgence and their well-being problems (H3). Specifically, compared with those in a less satisfying relationship, adolescents and their parents who are in a more satisfying relationship would be more vulnerable to the effect of disagreement in indulgent parenting on their well-being. 

## 2. Methods

### 2.1. Sample and Procedures

Adolescent participants were recruited from four high schools in the state of Florida in the United States. Both adolescents and their primary parents (the parent who does the most child-rearing) participated in this study, with parental consent and child assent duly obtained. Adolescent participants were tasked with completing an online survey (“student survey”) covering various topics, including their perceptions of their parents’ indulgent parenting behaviors, their own well-being concerns, and demographic information. Parent participants were asked to complete an online survey (“parent survey”) about their indulgent parenting behaviors, self-perceived well-being concerns, and demographic details. As a token of appreciation for their participation, each participant was compensated with $20 upon completing the questionnaires.

A total of 128 parent–adolescent dyads were included in this study. The adolescent participants in this study reported an average age of 15.22 years, ranging from 12 to 18 years old. The racial distribution among adolescents was 44% White, 44% African American, and 12% others. The majority of adolescent participants were non-Hispanic/Latino (83%) and 61% were female. Among parent participants, 57% reported within the age range of 40 to 49 years. Regarding racial and ethnic background, 51% were White, 42% were African American, and 7% were in other racial categories. The majority of parents identified as non-Hispanic or Latino (88%), were mothers (90%), and were either biological or adoptive parents (88%). Regarding educational attainment, 40% of the parents reported having at least a bachelor’s degree, with 24% having education levels higher than a bachelor’s degree. Regarding family income, the most frequently reported category for annual gross income was $25,000 to $50,000, while the median category was $75,000 to $100,000.

### 2.2. Measures

#### 2.2.1. Adolescents’ and Parents’ Perceptions of Indulgent Parenting

Both adolescents’ and their parents’ perceptions of indulgent parenting were assessed using a 30-item indulgent parenting scale, which has been used in prior studies and shown adequate reliability and validity [31]. Adolescents reported on both mother and father but only the report on the participating parent (primary parent) was included. Both adolescents and parents were asked to rate the 30 statements on a rating scale ranging from 1 (Strongly disagree) to 5 (Strongly agree). The scale had three dimensions with each dimension being assessed by 10 items: material indulgence, relational indulgence, and behavioral indulgence. Sample items included “my mother/father gives me all the shoes, accessories, and personal care items I want” (“I give him/her all the shoes, accessories, and personal care items he/she wants”) (material indulgence; α = 0.89 for adolescents and α = 0.72 for parents), “my mother/father is involved in everything I do” (“I am involved in everything he/she does”) (relational indulgence; α = 0.76 for adolescents and α = 0.70 for parents) and “my mother/father lets me get away without doing work she told me to do” (“I let him/her get away without doing work I told him/her to do”) (behavioral indulgence; α = 0.79 for adolescents and α = 0.79 for parents). Some items were reverse-coded. The items were then summed together for each dimension, with higher scores indicating higher levels of indulgence.

#### 2.2.2. Adolescents’ and Parents’ Well-Being Problems

The same measurements were administered to both adolescents and parents. Anxiety symptoms were assessed using a 10-item shortened version of the Beck Anxiety Inventory [32] (BAI). Participants were asked to indicate the extent to which they were affected by 10 common symptoms of anxiety over the past month, with response options ranging from 1 (not at all) to 4 (severely–it bothered me a lot). The scores from these items were summed, with higher scores indicating a greater level of anxiety (α = 0.89 for adolescents, α = 0.91 for parents). Stress levels were evaluated using seven items from the Rhode Island Stress and Coping Inventory [33]. Participants rated the frequency with each statement of their own life using a 5-point scale ranging from 1 (never) to 5 (frequently). The items were added up to represent participants’ stress levels over the past month (α = 0.89 for adolescents and α = 0.89 for parents). A higher score indicated a higher level of stress. Sample items included statements such as “I felt overwhelmed” and “I was pressured by others.” Life dissatisfaction was measured using the Satisfaction with Life Scale [34] (SWLS), which consisted of five statements (e.g., “The conditions of my life are excellent”). Participants were asked to rate their degree of agreement on a scale ranging from 1 (Strongly disagree) to 7 (Strongly agree) for each statement. All the scores were reverse-coded and then summed, with a higher total score indicating a higher level of life dissatisfaction (α = 0.87 for adolescents and α = 0.89 for parents). The three measures (anxiety, stress, and life dissatisfaction) were used as three indicators of latent constructs of well-being problems for adolescents and for their parents.

#### 2.2.3. Adolescents’ and Parents’ Relationship Satisfaction

Both adolescent and parent participants were asked the question, “How satisfied are you with your relationship with your mother/father/child?” which is a global assessment of relationship satisfaction. Participants reported their level of relationship satisfaction using a scale that ranged from 1 (Very unsatisfied) to 4 (Very satisfied).

#### 2.2.4. Covariates

Several covariates were considered in this study. Gender was coded as 1 for male and 2 for female. Race was categorized as 1 for White and 2 for non-White. Ethnicity was coded as 1 for Hispanic or Latino and 2 for Non-Hispanic or Latino. Education levels were dichotomized into two categories: 1 for high school graduates or lower and 2 for college/bachelor/post-bachelor’s degrees. Family structure was dichotomized as 1 for biological parents and 2 for other family arrangements. Family annual income was assessed across seven categories (i.e., below 25 k, 25 k to below 50 k, 50 k to below 75 k, 75 k to below 100 k, 100 k to below 150 k, 150 k to below 250 k, and 250 k and above).

### 2.3. Analytical Strategies

To test H1, we conducted three paired *t*-tests between adolescents’ and their parents’ reports, one for each dimension of indulgent parenting. To test H2, adolescents’ and their parents’ well-being problems, each as a latent construct, were regressed on the double-entry intraclass correlation coefficients (ICC_DE) [35,36] in the structural equation model (SEM). ICC_DE measures the profile similarity/agreement of indulgent parenting among adolescents and parents and is calculated as a Pearson correlation using the double-entry method [35,36]. Specifically, to compute ICC_DE, the values of adolescents’ reports of material, relational, and behavioral indulgence were entered first (and in that order), followed by parents’ material, relational, and behavioral indulgence (in that order). Then, the orders of adolescents’ reports and parents’ reports were flipped to create the double entries. ICC_DE scores were then calculated using the 12 original and transported entries. The advantage of ICC_DE, as compared to Pearson’s correlation, includes taking into account not just the shape of the data, but also the elevation and scatter [37]. To test H3, we created two interactions: ICC_DE and adolescents’ report of relationship satisfaction, and ICC_DE and parents’ report of relationship satisfaction. Adolescents’ and parents’ well-being problems were then regressed on the double-entry intraclass correlation coefficient (ICC_DE), adolescents’ and parents’ reports of parent–adolescent relationship satisfaction, and the two interaction terms. 

Based on previous research (e.g., [4,24]), we included several variables as covariates including adolescent gender, race, and ethnicity; and parent gender, race, ethnicity, family structure, education, and family income. In the preliminary models, we examined all these covariates. Only those covariates that showed significant effects were incorporated into the final model, ensuring a more parsimonious model.

## 3. Results

Table 1 provides descriptive information for the study variables and demographic information, including means, standard deviations, and proportions. From Table 1, one can see that, without exception, adolescents reported higher levels of indulgent parenting than their parents did. Results from paired *t*-tests (shown in Table 1) also suggested that the differences were statistically significant for all three dimensions of indulgent parenting (*p* < 0.01 for all), therefore supporting H1.

To test H2, the double-entry intraclass correlation coefficient score (ICC_DE) for each parent–child dyad was calculated. The mean of ICC_DE scores was 0.02 (SD = 0.58), indicating that adolescents’ and their parents’ perceptions of indulgent parenting were different in terms of scatter, shape, and slope. Table 2 shows the correlations among the key variables in the proposed model. The correlation matrix revealed several significant correlations between the double-entry intraclass correlation coefficient (ICC_DE) and psychological well-being problems for both adolescents and their parents. Specifically, ICC_DE was significantly and negatively correlated with adolescents’ reports of stress (*r* = −0.17, *p* < 0.05) and life dissatisfaction (*r* = −0.16, *p* < 0.05), as well as with parents’ reports of life dissatisfaction (*r* = −0.19, *p* < 0.05). The direction of the correlations between ICC_DE and adolescents’ anxiety and parents’ stress were also negative as expected. 

**Figure 1:**
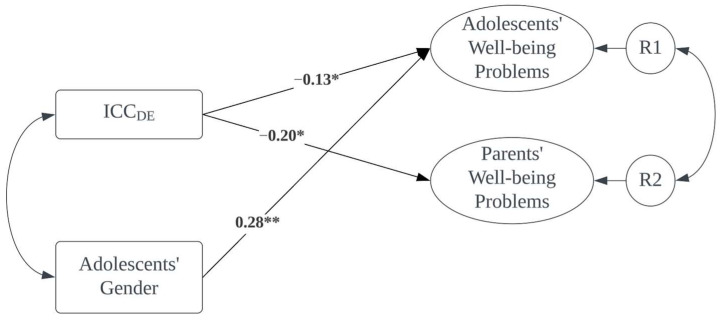
ICC_DE and parent–adolescent well-being problems. * *p* < 0.05, ** *p* < 0.01. Adolescent gender was included as a covariate.

The ICC_DE was then entered as a predictor of well-being outcomes in a structural equation model (SEM). We first included all the covariates in the model. Results suggested that adolescent gender was positively associated with adolescents’ well-being problems. No other covariates were significant. Therefore, adolescent gender was retained in the final models for subsequent analyses. The final model is shown in Figure 1. The model showed a good fit to the data: χ^2^ (18) = 26.4, *p* = 0.09; CFI = 0.94, RMSEA = 0.06, *p* close = 0.33. The paths from ICC_DE to well-being problems of both adolescents (β = −0.13, *p* < 0.05) and parents (β = −0.20, *p* < 0.05) were negative and significant, indicating that agreement between adolescents and their parents was negatively associated with well-being problems. In other words, disagreement regarding indulgent parenting between adolescents and their parents was related to higher levels of well-being problems for both adolescents and their parents, therefore supporting H2. Being female was associated with higher levels of well-being problems among adolescents (β = 0.28, *p* < 0.01).

**Figure 2:**
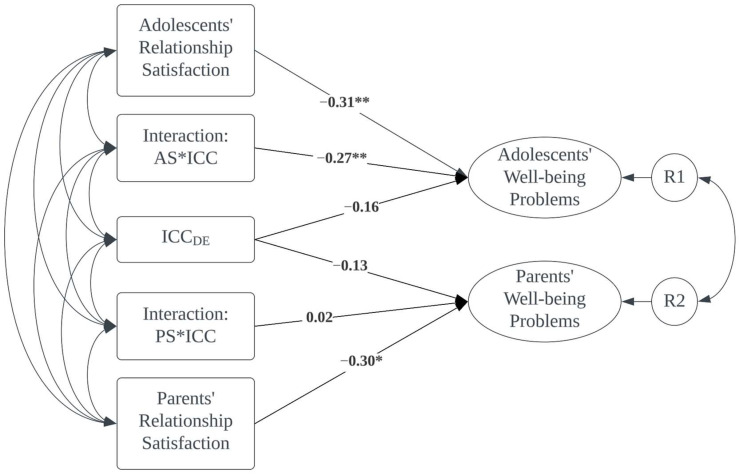
Relationship satisfaction as the moderator. * *p* < 0.05, ** *p* < 0.01. AS*ICC is the interaction between adolescent-reported relationship satisfaction and ICC; PS*ICC is the interaction between parent-reported relationship satisfaction and ICC.

To test H3, we regressed adolescents’ and parents’ well-being problems on ICC_DE, adolescents’ relationship satisfaction, parents’ relationship satisfaction, and the two interaction terms. Figure 2 illustrates the model. The model demonstrated a good fit to the data: χ^2^ (32) = 45.6, *p* = 0.06; CFI = 0.93, RMSEA = 0.06, *p* close = 0.34. The results showed a statistically significant interaction between adolescents’ relationship satisfaction and ICC_DE on their own well-being problems (β = −0.27, *p* < 0.01). A further look into the interaction suggested that, when comparing it with that of those less satisfied with their relationship with their parents, the association between disagreement in perceptions of indulgent parenting and adolescents’ well-being problems was stronger among adolescents who reported a more satisfying relationship with their parents. For parents, the interaction was not significant.

## 4. Discussion

Adolescence is a critical developmental stage with significant changes and challenges for both adolescents and their parents. Central to this phase is the pursuit of identity, independence, and autonomy, as highlighted by psychosocial development theories [7], the concept of developmental tasks [8], and self-determination theory [9]. Notably, indulgent parenting practices, which involve high responsiveness and low demandingness, have been associated with negative outcomes in adolescent development and psychological well-being, as indicated by multiple studies (e.g., [11,12,13]). These studies, however, often fail to consider the impact of indulgent parenting on the well-being of parents (e.g., [4,38]). Furthermore, due to the developmental tasks during adolescence, discrepancies often arise between how adolescents and their parents perceive parenting practices (e.g., [21]), such as degrees of indulgence, potentially giving rise to well-being issues. 

To address these questions, this study delves into the relationship between indulgent parenting and well-being problems within parent–adolescent dyads, recognizing both their differing perceptions in such parenting and the implications to their psychological well-being problems. We investigated discrepancies in how adolescents and their parents perceive indulgent parenting and explored the associations between these discrepancies and the psychological well-being challenges experienced by both adolescents and their parents. Additionally, we explored whether relationship satisfaction plays a role in these associations. Based on related theories and the literature, we hypothesized that adolescents’ perceptions of indulgent parenting would differ from those of their parents, with adolescents reporting higher levels of indulgent parenting (H1); discrepancies in parent–adolescent perceptions of indulgent parenting would be positively associated with psychological well-being problems for both adolescents and their parents (H2); and relationship satisfaction would moderate the relationship between parent–adolescent disagreement in indulgent parenting and their well-being problems. Results from this study generally supported these hypotheses.

### 4.1. Differences in Adolescents’ and Parents’ Perceptions of Indulgent Parenting (H1)

This developmental stage of adolescence warrants a renegotiation of the appropriate levels of parental involvement and control, aligning with the concept of achieving a “goodness of fit” [15,16]. Importantly, during this period, adolescents may perceive parenting they receive differently from how their parents perceive their own parenting practices [21]. This evolving dynamic reflects the unique challenges and transformations that both adolescents and their parents experience as they navigate this critical developmental juncture.

Consistent with related theories and research, results from paired *t*-tests supported our hypothesis that adolescents perceived higher levels of indulgent parenting than their parents did. With their pursuits of independence and the identity of self, adolescents may perceive indulgent parenting behaviors as preventing them from achieving these pursuits. Therefore, they might be more sensitive to and aware of the degree of indulgent parenting from their parents than their parents do. The results showed that adolescents perceived a significantly higher degree of indulgent parenting in all three dimensions than their parents did. While parents may perceive that they provide the appropriate amount of indulgence to their adolescent children, their adolescent children feel their parents are overly responsive while also lacking behavioral reinforcement at this developmental stage. The finding adds to the findings from limited existing studies that there were systematic differences and discrepancies between parents’ and their children’s reports of parenting behaviors [19,20] and with the findings on indulgent parenting [23,24]. 

### 4.2. Parent–Adolescent Disagreement over Indulgent Parenting and Their Well-Being (H2)

As suggested by the stage–environment fit theory [18], the mismatch between parental indulgence practices and the needs of developing adolescents, as reflected by discrepancies in perceptions of indulgent parenting, could bring negative effects on the well-being of both adolescents and their parents. ICC_DE analyses in this study provided supporting evidence for our hypothesis (H2). We found that disagreement among adolescents and their parents regarding indulgent parenting was associated with more well-being problems, which supported the notion that failure in achieving the goodness of fit and a low fit between parenting and adolescent development could produce negative impacts on adolescents’ development and adjustment [15,16,17]. 

Our previous study using the same dataset suggested that adolescents’ and their parents’ perceived indulgence was related to their own well-being problems [23]. We did not, however, find their perceptions of indulgence were related to each other’s well-being problems in that study. Such findings beg the question of whether there were differences in their perceptions of indulgence. Differences between parents’ and adolescents’ perceptions of indulgent parenting may be meaningful when relating to the psychological well-being of both adolescents and their parents. The perspectives from both parents and adolescents provide valid information [39] and the disagreement among them could reflect different attitudes and beliefs. Most previous research did not conduct dyadic analyses or take both of their perspectives into account when examining indulgent parenting behaviors. We utilized a dyadic approach and found that disagreement in perceptions of indulgent parenting was related to the psychological well-being problems of both adolescents and their parents. 

No previous studies have addressed the relationship between parent–adolescent discrepancies of indulgent parenting and both parents’ and adolescents’ well-being problems. As suggested by the association between indulgent parenting and the well-being problems of parents of emerging adult children, parents could also be affected by indulgent parenting [4]. Parents who practice indulgent parenting could spend a lot of time and resources trying to satisfy their children’s needs and wants and to shield them from the consequences of their misbehaviors. When such effort is perceived negatively by their children (e.g., unwanted), this could become the source of their well-being problems (e.g., stress and dissatisfaction). The finding, thus, addressed the importance of communicating and renegotiating developmentally appropriate parenting behaviors between parents and their adolescent children for the well-being of both adolescents and their parents. 

Our analysis revealed a significant relationship between adolescent gender and their well-being. Specifically, the results indicated that adolescent girls reported higher levels of well-being problems compared to their male counterparts. This finding aligns with prior research that has consistently demonstrated that adolescent girls exhibit a heightened vulnerability to various psychological difficulties, including intrafamilial stress and depressive symptoms [40,41]. It underscores the importance of recognizing and addressing the unique challenges and well-being concerns that adolescent girls may encounter during their development.

### 4.3. Parent–Adolescent Relationship Satisfaction as a Moderator (H3)

Existing research consistently highlights the positive role of parent–adolescent relationship satisfaction in the psychological well-being of adolescents, emphasizing the benefits of a positive and satisfying parent–child relationship (e.g., [6,30]). The results of our study, however, revealed something interesting. We found that if adolescents were more satisfied with their relationship with their parents, disagreement over their perceptions of indulgent parenting was more strongly related to their well-being problems. It is possible that, in relationships characterized by higher satisfaction, parent–adolescent disagreement carries more emotional weight on the adolescents, leading to their heightened stress and psychological well-being challenges. We did not find the same effect for parents. 

Adolescence, characterized by identity formation, the pursuit of independence, and the continuing need for parental support and guidance [7], underscores the significance of agreement with parents, especially regarding perceptions of parenting practices. Such agreement aids in adolescents’ self-concept development and minimizes internal conflict. Adolescents in satisfying relationships may hold higher expectations for parental agreement in their interactions, and when these expectations are unmet, it could lead to negative emotions and a deficiency in fulfilling their needs, which, in turn, relates to psychological well-being issues. Therefore, open communication, mutual understanding, and collaborative resolution of disagreement regarding developmental issues remain crucial for both adolescents and parents [15,23].

### 4.4. Limitations and Future Research

The present study has several limitations that should be considered when interpreting the findings. First, the sample size was relatively small, lacked diversity in some demographic characteristics, and predominantly consisted of mothers as participating parents. The study’s single location in the state of Florida limited its generalizability. Traditional parenting beliefs and practices could differ by region in the U.S., and a sample from Florida is not representative of parents and adolescents from other states in the South or the broader U.S. [42]. Further, previous research has highlighted the importance of fathers in parenting (e.g., [43,44]), underscoring the need for future studies to employ larger and more diverse samples to validate the current findings. In addition, future research should look into the possible differences in mothers’ and fathers’ indulgent parenting to their sons and daughters. Second, we used a cross-sectional design, which limits our ability to further delineate the directionality of associations. While this study proposed and tested a unidirectional model based on existing theories and research, a longitudinal approach would be necessary to explore potential bidirectional impacts and gain deeper insights into the relationships between indulgent parenting and well-being. Third, our study only examined discrepancies in adolescents’ and parents’ perceived indulgence; therefore, findings could not be generalized to other parenting behaviors. However, because it is possible that parent–adolescent disagreement could be a source of stress for both of them regardless of the topics, future studies should further examine whether parent–adolescent disagreement and their association with well-being are parenting behavior-specific. Finally, this study only investigated the moderating role of parent–adolescent relationship satisfaction; future studies should further explore the various potential mechanisms explaining the bivariate association between disagreement in indulgent parenting and well-being problems among adolescents and their parents, such as communication and conflicts between parents and adolescents [45,46]. 

### 4.5. Implications

Despite the limitations, the present study extended the current limited research by addressing the importance of indulgent parenting and filling a gap by using the dyadic approach to understand the different perceptions between parents and adolescents. This study also indicated the role of indulgence practice in parents’ own psychological well-being problems in addition to adolescents’ well-being problems. Therefore, it is important for adolescents and parents to renegotiate indulgent parenting behavior at this particular time of adolescence, according to adolescents’ developmental needs, which benefits both adolescents and parents. 

The findings from our study could offer valuable insights for practitioners working with adolescents and their families. It is informative for parent education programs to include components that educate both parents and adolescents about the process of self-development and the pursuit of independence during adolescence. By fostering awareness of these developmental shifts, practitioners could facilitate better understanding and communication between parents and adolescents. Encouraging open dialogue between adolescents and their parents to address the evolving needs of adolescents is essential, ultimately leading to a more harmonious and mutually agreeable approach to developmentally appropriate parenting behaviors. Facilitating timely and transparent communication between parents and adolescents could contribute to the cultivation of a shared perspective on parental involvement. This, in turn, can help mitigate discrepancies and disagreement regarding perceptions of parenting practices and align more closely with the concept of achieving a “goodness of fit” [15,16]. Taken together, these strategies can enhance the quality of parent–adolescent relationships, promote effective parenting practices, and support the well-being of all family members, especially adolescents, during this critical phase of development.

## Figures and Tables

**Table 1:** Descriptive and demographic information on study variables and paired *t*-test results.

Variables	Adolescents		Parents	
	M (%)	S.D.	M (%)	S.D.
Indulgent Parenting				
Material Indulgence	29.59 ^a^	7.72	24.20 ^a^	5.03
Relational Indulgence	27.74 ^b^	5.93	25.51 ^b^	4.93
Behavioral Indulgence	22.01 ^c^	5.74	19.71 ^c^	4.93
Psychological Well-being Problems				
Anxiety	18.21	6.80	14.73	5.57
Stress	19.19	6.92	18.86	5.83
Life Dissatisfaction	17.19	7.11	16.44	6.59
Relationship Satisfaction	3.38	0.93	3.49	0.81
Demographics				
Age	15.22	1.12	40–49 (57%)	
White	44%		44%	
Female	61%		90%	
Non-Hispanic or Latino	83%		88%	
Biological or Adoptive Mothers			88%	
Bachelors and Higher			64%	
Family Annual Income (below 100k)			69%	
ICC M (S.D.)	0.02 (0.58)			

Note. *n* = 128 dyads. ^a, b, c^: significant differences between the adolescent and parental reports of indulgent parenting (*p* < 0.01 in paired *t*-test).

**Table 2:** Correlations for study variables.

Variables	1	2	3	4	5	6	7	8	9
1. ICC_DE	1.00								
Adolescents									
2. Anxiety	−0.12	1.00							
3. Stress	−0.17 *	0.65 **	1.00						
4. Life Dissatisfaction	−0.16 *	0.33 **	0.28 **	1.00					
5. Relationship Satisfaction	0.11	−0.21 *	−0.18 *	−0.38 **	1.00				
Parents									
6. Anxiety	0.06	0.09	0.02	−0.01	−0.17 *	1.00			
7. Stress	−0.15	0.18 *	0.20 *	0.07	−0.18 *	.43 **	1.00		
8. Life Dissatisfaction	−0.19*	0.07	0.06	0.17 *	−0.13	0.29 **	0.34 **	1.00	
9. Relationship Satisfaction	0.05	−0.03	−0.13	−0.22 *	0.33 **	−0.24 **	−0.17 *	−0.19 *	1.00

Note. *n* = 128 dyads. * *p* < 0.05, ** *p* < 0.01.

## Data Availability

The data presented in this study are available on request from the corresponding author. The data are not publicly available due to privacy.
